# Bleeding From a Lateral Duodenal Diverticulum Complicated by a Retroperitoneal Perforation After Endoscopic Clipping

**DOI:** 10.1155/crgm/9493004

**Published:** 2026-10-17

**Authors:** Páll Guðjónsson, Hallgrímur Guðjónsson, Einar Stefán Bjornssön

**Affiliations:** ^1^ Landspitali–The National University Hospital of Iceland Reykjavik Iceland; ^2^ Faculty of Medicine University of Iceland Reykjavik Iceland hi.is

## Abstract

Duodenal diverticula (DD) are commonly encountered in the periampullary region but rarely on the lateral wall of the duodenum. They can develop complications such as diverticulitis, bleeding, and perforation. A 69‐year‐old healthy woman presented with melena due to a bleeding lesion in a lateral DD that was treated with hemoclipping. Two days later, she developed fever, hypotension, and an elevated CRP with mild abdominal pain and had no signs of peritonitis. A CT scan with oral contrast showed a large diverticulum at the junction of the descending and horizontal part of the duodenum and a perforation with minimal clinical symptoms that was successfully treated conservatively. Although causality could not be established, a retroperitoneal perforation following endoscopic hemostasis was a suspected complication of hemostatic clipping. Physicians should have a low threshold for a CT scan in the case of raised fever or inflammatory markers following endoscopic management of DD.

## 1. Introduction

Duodenal diverticula (DD) are relatively common, with a prevalence of around 4%–22% based on autopsy results [1, 2]. DD are found incidentally in around 10%–30% of ERCPs, mainly peri‐ampullary, and increase with age [3–5]. The majority remain asymptomatic, but complications related to DD have been reported in around 1%–5% and include diverticulitis, bleeding, perforation, and pancreatitis [6, 7]. Perforation of a DD into the retroperitoneum is a serious complication that carries a risk of mortality from 5% to 30% [8–10]. The most common causes of perforation are diverticulitis (62%), enterolithiasis (10%), iatrogenic (5%), ulceration (5%), and various rarer causes [9]. Only two case reports of perforation after endoscopic treatment of a bleeding DD with hemoclip exist [11, 12], and another after argon plasma coagulation [13]. These cases, together with the current case, suggest that perforation should be considered as a potential complication of endoscopic management of DD.

## 2. Case Report

A 69‐year‐old previously healthy woman, taking 75 mg of aspirin daily, presented to the emergency department with melena for the last 10 days. On arrival, her hemoglobin level was 77 g/L. She received 2 units of packed red blood cells and parenteral esomeprazole. She was afebrile, had mild RUQ tenderness without signs of peritonitis, her CRP levels were < 3 mg/L, and the white blood cell count was normal. A forward‐viewing esophagogastroduodenoscopy (EGD) using room air for insufflation was performed and showed a division of the duodenum beyond the duodenal bulb. On the left side was the proper lumen and to the right a large diverticulum (Figure 1). Inside the diverticulum were signs of active bleeding from a small mucosal laceration, or tear, which did not look like a typical peptic ulcer (Figure 2). Hemostasis was achieved by applying two hemoclips directly to the bleeding lesion without any tissue capture maneuvers (Figure 3). A diagnosis of a DD was made, based on typical endoscopic findings with a mucosa‐lined epithelium connected directly with the duodenal lumen. Two days after presentation and the endoscopy, she developed a fever of 38.3°C and hypotension. A follow‐up endoscopy was performed that found the clips still in place and no signs of bleeding (Figure 4). New blood tests showed an elevated CRP of 80 mg/L, and the fever elevated to 38.7°C. A CT scan with oral contrast was performed for further work‐up, which showed a 3.3 × 3.5 cm diverticulum at the junction of the descending and the horizontal part of the duodenum (Figure 5) and free air around the diverticulum and Gerota’s fascia, indicating retroperitoneal perforation of the duodenum. Surgery was consulted, and since there were no signs of peritonitis, no extravasation of oral contrast, and the patient was hemodynamically stable after fluid resuscitation, they advised conservative management. In addition, there were no intraprocedural signs suggestive of perforation during the endoscopies. The patient was kept fasting for 2 days and received parenteral ceftriaxone and metronidazole for 3 days with rapid resolution of the fever. At that time, she tolerated an oral diet well and was discharged with 4 days of oral amoxicillin/clavulanic acid treatment. Two months later, she was evaluated by a general surgeon who found no indication for surgery. Over a 5‐year follow‐up period, there were no signs of recurrent bleeding.

**FIGURE 1:**
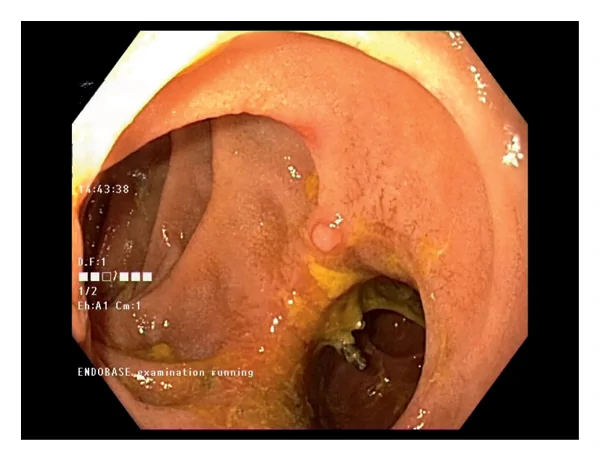
Diverticulum orifice on the right side and a normal lumen on the left side.

**FIGURE 2:**
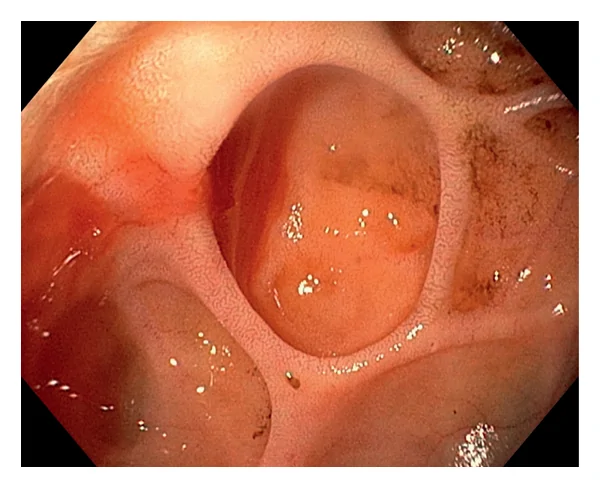
The bleeding lesion before treatment.

**FIGURE 3:**
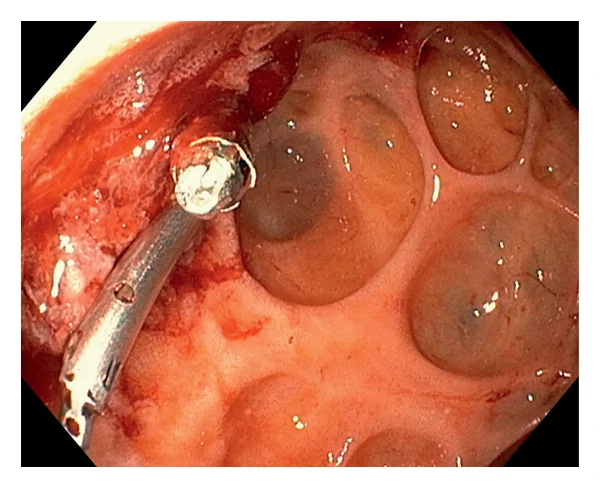
Hemostatic treatment with hemoclip application.

**FIGURE 4:**
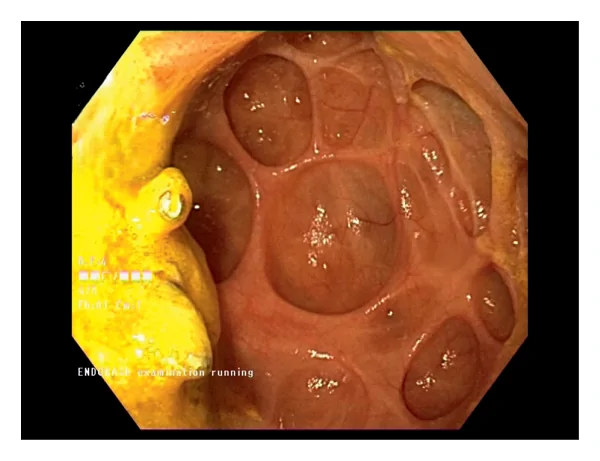
A follow‐up endoscopy 2 days after presentation showing the inside of the diverticulum with the hemoclip still in place.

**FIGURE 5:**
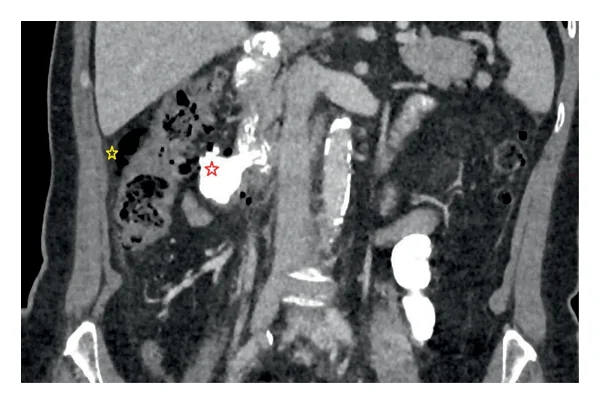
A CT scan with oral contrast. It shows the duodenal diverticulum marked with a red star. It measured 3 × 3 × 3.5 cm. Free air is around the diverticulum. More free air is demonstrated in front of Gerota’s fascia and behind the colon, marked with a yellow star.

## 3. Discussion

This case report describes two rare complications of a large and atypical DD: bleeding and retroperitoneal perforation, the latter suspected to be a complication after endoscopic hemostasis with clipping. The DD, being laterally located, is at an unusual site, as most DD’s are periampullary and on the medial side of the duodenum. According to autopsy studies, the most common site for a duodenal diverticulum is the second part of the duodenum (62%), followed by the third (27%) and the fourth parts (8%) [2]. Only 5% of DD are found on the lateral part [7]. However, since no histopathological confirmation was made in the present case, it limits conclusions regarding the exact nature and wall structure of the diverticulum. The majority of DD are acquired extraluminal diverticula that consist only of mucosa and submucosa but lack the muscularis propria, like a normal bowel wall [8], making it more vulnerable. Clipping this thin wall might grasp the entire wall thickness, which could lead to localized ischemia and delayed perforation through pressure necrosis. It might also be more sensitive to traction generated from clipping and peristalsis and could be more susceptible to barotrauma during insufflation. Although the timeline suggests delayed perforation after endoscopic management, it is also conceivable that the bleeding lesion reflected a mucosal injury that led to perforation prior to the endoscopy. During endoscopic procedures within a DD, a cautious approach using as few hemoclips as possible and CO2 [14] rather than room air could be considered to minimize the risk associated with the procedure. Endoscopic clipping is a very safe procedure [15]. Perforation of a DD has also been described following argon plasma coagulation [13], and there is no evidence to suggest alternative hemostatic modalities than clipping in patients with DD.

Based on nonrandomized studies, conservative treatment of perforated DDs is preferred over surgical management because of lower complication rates (28% vs. 4%) and mortality (8% vs. 0%) [10]. These numbers might be affected by selection bias since severely ill patients are more likely to undergo surgery. Despite that limitation, a conservative approach should be considered for clinically stable patients with close monitoring and reconsideration for surgery in the case of clinical deterioration.

Hemoclipping is considered a very safe endoscopic treatment of a bleeding ulcer [15], and reports of perforation following clip application are rare. Nevertheless, physicians should consider it and have a low threshold for a CT scan in patients who develop fever or elevated inflammation markers in the days following the endoscopic management of DD even without severe abdominal pain or signs of peritonitis.

## Funding

No funding was received for this study.

## Consent

Written informed consent was obtained from the patient for publication of clinical information and images.

## Conflicts of Interest

The authors declare no conflicts of interest.

## Data Availability

The data that support the findings of this study are available on request from the corresponding author. The data are not publicly available due to privacy or ethical restrictions.
